# Characterization of gene expression on genomic segment 7 of infectious salmon anaemia virus

**DOI:** 10.1186/1743-422X-4-34

**Published:** 2007-03-29

**Authors:** Frederick SB Kibenge, Hongtao Xu, Molly JT Kibenge, Biao Qian, Tomy Joseph

**Affiliations:** 1 Department of Pathology and Microbiology, Atlantic Veterinary College, University of Prince Edward Island, 550 University Avenue, Charlottetown, P.E.I., C1A 4P3, Canada; 2 McGill AIDS Centre, Jewish General Hospital, 3755 Cote Ste Catherine Road, Montreal, Quebec, H3T 1E2, Canada; 3 Laboratory of Infectious Diseases, NIAID, NIH, Bldg 50, Room 6132 50 South Drive, MSC 8007, Bethesda, MD 20892, USA

## Abstract

**Background:**

Infectious salmon anaemia (ISA) virus (ISAV), an important pathogen of fish that causes disease accompanied by high mortality in marine-farmed Atlantic salmon, is the only species in the genus *Isavirus*, one of the five genera of the *Orthomyxoviridae *family. The *Isavirus *genome consists of eight single-stranded RNA species, and the virions have two surface glycoproteins; haemagglutinin-esterase (HE) protein encoded on segment 6 and fusion (F) protein encoded on segment 5. Based on the initial demonstration of two 5'-coterminal mRNA transcripts by RT-PCR, ISAV genomic segment 7 was suggested to share a similar coding strategy with segment 7 of influenza A virus, encoding two proteins. However, there appears to be confusion as to the protein sizes predicted from the two open reading frames (ORFs) of ISAV segment 7 which has in turn led to confusion of the predicted protein functions. The primary goal of the present work was to clone and express these two ORFs in order to assess whether the predicted protein sizes match those of the expressed proteins so as to clarify the coding assignments, and thereby identify any additional structural proteins of ISAV.

**Results:**

In the present study we show that ISAV segment 7 encodes 3 proteins with estimated molecular masses of 32, 18, and 9.5 kDa. The 18-kDa and 9.5-kDa products are based on removal of an intron each from the primary transcript (7-ORF1) so that the translation continues in the +2 and +3 reading frames, respectively. The segment 7-ORF1/3 product is variably truncated in the sequence of ISAV isolates of the European genotype. All three proteins are recognized by rabbit antiserum against the 32-kDa product of the primary transcript, as they all share the N-terminal 22 amino acids. This antiserum detected a single 35-kDa protein in Western blots of purified virus, and immunoprecipitated a 32-kDa protein in ISAV-infected TO cells. Immunofluorescence staining of infected cells with the same antiserum revealed the protein(s) to be localized in the cytoplasm. Vaccination of farmed Atlantic salmon with the 32-kDa protein resulted in a higher survival rate than what was attainable with the HE protein, albeit a moderate protection against the low ISAV challenge.

**Conclusion:**

Collectively, our observations suggest that the product of ISAV segment 7 primary transcript (7-ORF1) is a structural protein. The 18-kDa (7-ORF1/2) protein is identified as the putative ISAV nuclear export protein based on the presence of nuclear export signals. The function of the 9.5-kDa (7-ORF1/3) protein is not presently known.

## Background

Infectious salmon anaemia (ISA) virus (ISAV) is an important pathogen of fish that causes disease accompanied by high mortality in marine-farmed Atlantic salmon. The disease has caused severe economic losses to the salmon-farming industry in several countries in the northern hemisphere including Norway, Faroes Islands (Denmark), USA and Canada, and confirmed positive diagnostic results for ISA are reportable to the "Office International des Epizooties", OIE [1]. It is generally accepted that vaccination is the best option for control of the disease in marine-farmed fish, but the presently available whole virus inactivated vaccine that is used in Maine, USA, and Canada does not fully protect against the virus [2].

ISAV is the only species in the genus *Isavirus*, which is one of five genera of the *Orthomyxoviridae *family [3]. Viruses in the genus *Isavirus *contain a segmented genome of eight single-stranded RNA species of negative sense ranging in length from 1.0 to 2.4 kb with a total molecular size of approximately 14.3–14.5 kb [4,5]. Different researchers have collectively sequenced all eight RNA genomic segments of ISAV. Assignments of viral proteins to these segments have been predominantly based on amino acid composition, computer-assisted motif identification, and counterparts of influenza viruses. It is becoming increasingly clear that the gene-coding assignments of the ISAV genome differ from those of other orthomyxoviruses [1,6]. Thus the enveloped particles have a diameter of 90–140 nm with two surface glycoproteins; haemagglutinin-esterase (HE) protein, a receptor binding haemagglutinin with receptor destroying enzyme activity demonstrated to be an acetylesterase [7,8] encoded on segment 6, and fusion protein (F) [9] encoded on segment 5. Sequence analysis of several ISAV isolates on the eight segments consistently reveals two genotypes, one European and one North American.

The ISAV segment 7 was originally assumed to encode two putative matrix proteins [10], analogous to M1 and M2 proteins of segment 7 of influenza A virus [11]. In that proposed gene expression model for ISAV segment 7, the putative M2 protein shared the first 22 amino terminal residues including the initiating methionine of the M1, but with the removal of the 526 nucleotide intron and resultant frameshift, the translation continued in the +2 reading frame terminating downstream of the M1 exon [10]. However, the M1 and M2 proteins predicted to have molecular weights of 34.1 kDa and 17.7 kDa, respectively, were not verified, although M2 was suggested as the fusion protein because of its similarity (38% identity) to the fusion glycoprotein F of rinderpest virus [10]. Biering *et al*. [12] expressed segment 7 open reading frame (ORF) 1 in *Escherichia coli *and prepared rabbit antibodies to the expressed protein. Because the segment 7 ORF1 protein did not react with whole ISAV antiserum, it was considered to be nonstructural or a minor structural protein [12]. In subsequent studies, these authors reported the 7-ORF1 product to be an interferon-signalling antagonist, a function similar to that of the non-structural (NS1) protein encoded by segment 8 of influenza A virus [13]. It is apparent that the structural protein profile of ISAV has not been conclusively determined. This information is essential in order to understand the viral proteins that are important in inducing protective immune responses in fish and/or viral proteins that could be targeted in viral diagnosis.

The primary goal of the present work was to clone and express the two ORFs in ISAV segment 7 in order to assess whether the protein sizes predicted from the ORFs match those of the expressed proteins so as to clarify the coding assignments, and thereby identify any additional structural proteins of ISAV.

## Results and Discussion

### *In-vitro *expression of ISAV proteins

**Figure 1:**
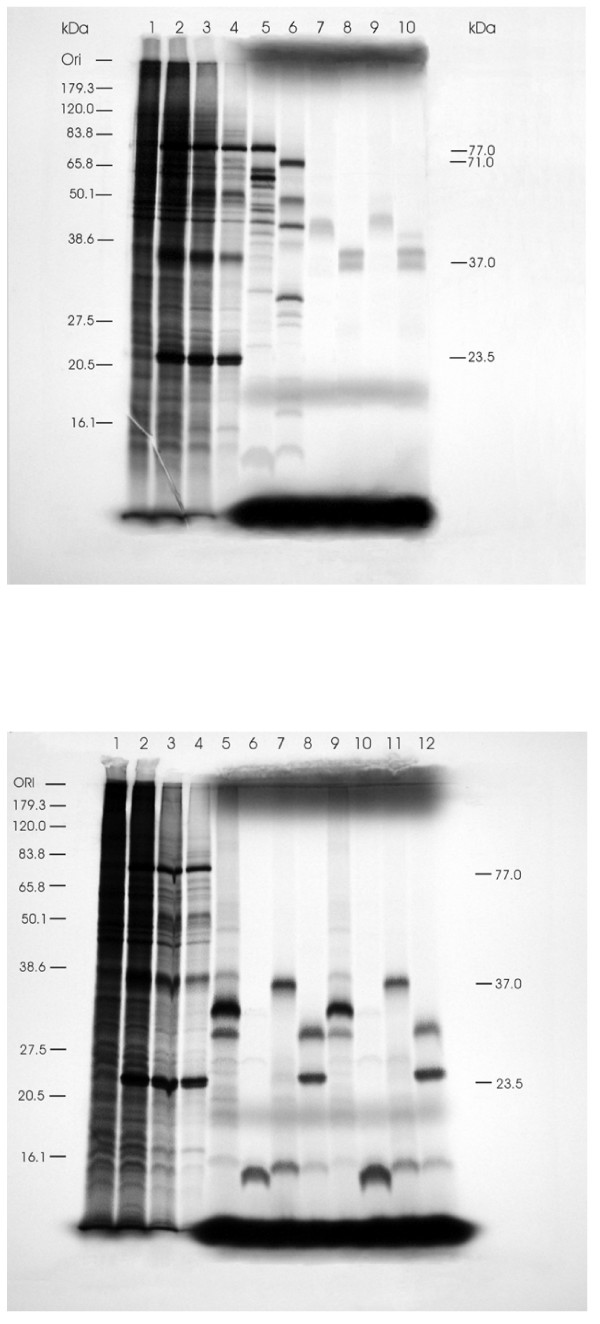
**Autoradiograph of SDS-PAGE of expressed proteins labeled with [^35^S]methionine in a coupled *in vitro *TNT System with the different constructs in pCR^®^II-TOPO^® ^plasmid and in TO cells infected with ISAV**. The prestained protein standards (Invitrogen Life Sciences) are drawn in on the left while corresponding molecular masses of the ISAV proteins seen above the background of cellular proteins are indicated on the right. **(A) **Lanes 1 to 8 contain radiolabeled proteins synthesized in uninfected TO cells, ISAV-infected TO cells at 24, 48 and 96 hr post-infection, and in TNT System with constructs of ORFs in ISAV segments 3–6, respectively. Lanes 9 and 10 contain radiolabeled proteins synthesized in TNT System in presence of canine microsomes with constructs of ORFs in ISAV segments 5 and 6, respectively. A band correlating with the product of segment 4 was visible in ISAV-infected cells at 96 hrs (lane 4), and is therefore indicated on the right. **(B) **Lanes 1 to 8 contain radiolabeled proteins synthesized in uninfected TO cells, ISAV-infected TO cells at 24, 48 and 96 hr post-infection, and in TNT System with constructs of ISAV segment 7 ORFs 1 and 2 and segment 8 ORFs 1 and 2, respectively. Lanes 9 to 12 contain radiolabeled proteins synthesized in TNT System in presence of canine microsomes with constructs of ISAV segment 7 ORFs 1 and 2 and segment 8 ORFs 1 and 2, respectively.

All the eight RNA genomic segments of ISAV have been sequenced, but it is not known if the protein sizes predicted from all the ORFs match those of the expressed proteins or if all the viral structural proteins of ISAV can be accounted for. In particular, there appears to be confusion as to the protein sizes predicted from two ORFs of segment 7 which has in turn led to confusion of the predicted protein functions. For example, Biering *et al*. [12] were unable to identify a protein of the size predicted by ORF1, the largest ORF of segment 7, in Western blots using whole ISAV antiserum, leading them to conclude that it was a non-structural viral protein. To address this question, 10 different constructs representing the known ORFs in the eight RNA genomic segments (1 each for RNA segments 1–6 and 2 each for RNA segments 7 and 8) were made in pCR^®^II-TOPO^® ^vector (Table 1). They were then expressed in an *in vitro *T7/SP6 coupled transcription-translation system and the gene products were correlated to metabolically labeled ISAV proteins synthesized in ISAV-infected cells. Protein products were monitored by SDS-PAGE as shown in Figure 1, which represents a direct comparison between the *in vitro *translation products and the proteins synthesized in ISAV-infected TO cells. Three proteins which appeared as prominent bands above the background bands of cellular proteins of ISAV-infected cell lysates (77 kDa, 37 kDa and 23.5 kDa) (Fig. 1A and 1B) were unambiguously assigned to viral RNA segments 3, 6, and 8 ORF2, and corresponded to nucleoprotein (NP), HE, and matrix (M) proteins, respectively. A 71 kDa band correlating with the product of segment 4 (lane 6) was also weakly visible in ISAV-infected cells at 96 hr (lane 4), hence the mark on Figure 1A. Our results confirmed that the observed molecular mass of the HE protein in ISAV-infected cells is 37 kDa (Fig. 1A, lanes 2–4), as reported previously by Griffiths *et al*. [14]. The HE protein in purified virus particles has higher molecular mass [7] probably resulting from post-translational modifications such as glycosylation.

**Table 1:** The oligonucleotide primers used in amplification of viral cDNA and construction of transcription vectors for ISAV genes

Designated name and (size) of primer	Sequence	Nucleotide position, and GenBank Accession number	
ISAV SEG1p1 FOR (26 mer)	GTCGACCAGCTAAGAATGGACTTTAT	1–20,	AY168787
ISAV SEG1p4 REV (28 mer)	GGTACCTTAAACACCATATTCATCCATC	1634–1685,	AF404347
ISAV SEG2p5 FOR (28 mer)	GTCGACATAACCATGGAAACTCTAGTAG	29–50,	AF404346
ISAV SEG2p8 REV (28 mer)	GGTACCTCAAACATGCTTTTTCTTCTTA	2140–2161,	AF404346
ISAV SEG3 FOR (18 mer)	ATGGCCGATAAAGGTATG	49–66,	AF404345
ISAV SEG3 REV (18 mer)	CTAAATGTCAATGTCTTC	1882–1899,	AF404345
ISAV SEG4 FOR (20 mer)	ATGGATAACCTCCGTGAATG	5–24,	AF404344
ISAV SEG4 REV (18 mer)	TCATTGGGTACTGACTGC	1724–1741,	AF404344
ISAV SEG5 FOR (21 mer)	ATGGCTTTTCTAACAATTTTA	9–29,	AF404343
ISAV SEG5 REV (21 mer)	CTACCCTTTAGTAATGTACAG	1368–1388,	AF404343
ISAV SEG6 FOR (25 mer)	ATGGCACGATTCATAATTTTATTCC	20–44,	AJ276859
ISAV SEG6 REV (24 mer)	GTTGTCTTTCTTTCATAATCAAGC	1181–1204,	AJ276859
ISAV SEG7 (ORF1) FOR (23 mer)	ATGGATTTCACCAAAGTGTATGG	22–44,	AF328627
ISAV SEG7 (ORF1) REV (23 mer)	TCACATTCTGAAGTGAAGTCCAG	902–924,	AF328627
ISAV SEG7 (ORF2) FOR (82 mer)	ATGGATTTCACCAAAGTGTATGGT	22–84/611–629,	AF328627
	GTGCTGGTTGACCAACTAAAACTT		
	CACGGAAAAGACAAGGTGGCTTCT TTCCTGTCGG		
ISAV SEG7 (ORF2)_REV (24 mer)	CATCTTTAGTTCTCATTACAAATG	1009–1032,	AF328627
ISAV SEG8 (ORF1) FOR (18 mer)	ATGAACGAATCACAATGG	12–29,	AF312317
ISAV SEG8 (ORF1) REV (18 mer)	TTACTTCAGGTACCCCAG	585–602,	AF312317
ISAV SEG8 (ORF2) FOR (21 mer)	ATGGATACAAAAACATCTACC	26–46,	AF312317
ISAV SEG8 (ORF2) REV (21 mer)	ATTTATTGTATAGAGTCCTCC	733–753,	AF312317

The underlined sequences in segments 1 and 2 primers correspond to *Sal *I site: GTCGAC and *Kpn *I site: GGTACC

**Figure 2:**
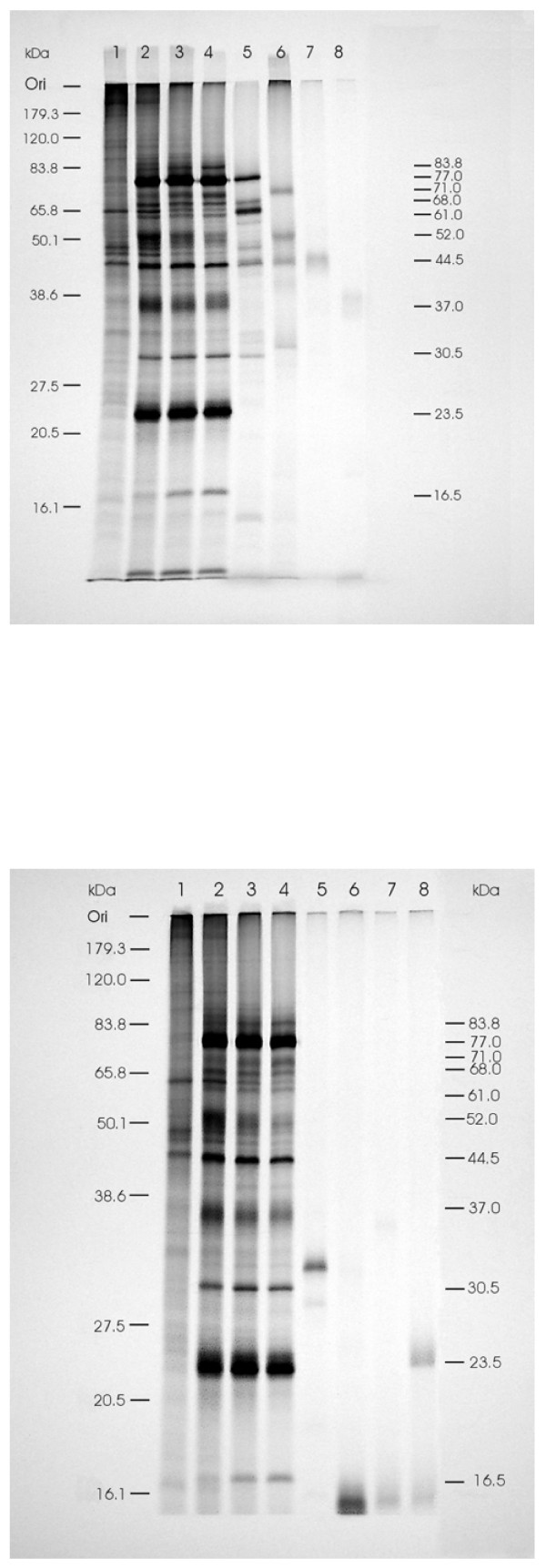
**Autoradiograph of SDS-PAGE of expressed proteins labeled with [^35^S]methionine in a coupled *in vitro *TNT System with the different constructs in pCR^®^II-TOPO^® ^plasmid and in TO cells infected with ISAV as in Fig. 1, immunoprecipitated with rabbit antiserum to purified ISAV**. The prestained protein standards (Invitrogen Life Sciences) are drawn in on the left while corresponding molecular masses of immunoprecipitated ISAV proteins are indicated on the right. **(A) **Lanes 1 to 8 contain radiolabeled proteins synthesized in uninfected TO cells, ISAV-infected TO cells at 24, 48 and 96 hr post-infection, and in TNT System with constructs of ORFs in ISAV segments 3–6, respectively. **(B) **Lanes 1 to 8 contain radiolabeled proteins synthesized in uninfected TO cells, ISAV-infected TO cells at 24, 48 and 96 hr post-infection, and in TNT System with constructs of ISAV segment 7 ORFs 1 and 2 and segment 8 ORFs 1 and 2, respectively.

To eliminate the background bands in ISAV-infected cell preparations and *in vitro *translation products and identify all the structural viral proteins, we carried out immunoprecipitation analysis with rabbit antiserum against purified whole ISAV. Analysis of the immunoprecipitates revealed up to six major protein bands in ISAV-infected TO cells consisting of the 77 kDa, 44.5 kDa, 37 kDa, 30.5 kDa, 23.5 kDa, and 16.5 kDa proteins, with the most intense bands being of the 77 kDa and 23.5 kDa proteins. The 77 kDa, 44.5 kDa, 37 kDa, and 23.5 kDa proteins co-migrated with the gene products of segments 3, 5, 6, and 8 ORF2, respectively (Figs 2A and 2B), which in purified ISAV correspond to NP, F, HE, and M proteins, respectively. The 30.5 and 16.5 kDa proteins in immunoprecipitates of ISAV-infected TO cells corresponded to segment 7 ORFs 1 and 2 products, of 32.5 and 16.0 kDa respectively, which were also immunoprecipitated with whole ISAV antiserum (Fig. 2B). Indeed in gels ran with different sets of protein molecular weight standards, the corresponding immunoprecipitates of ISAV-infected TO cells were 32 and 18 kDa, respectively, indicating that one or both segment 7 products are components of ISAV virions. We previously reported a 29–30 kDa protein which was immunoprecipitated in different infected TO cell lysates for 5 different ISAV strains using the same antiserum against purified ISAV as in the present study [15]. But others reported the segment 7 ORF 1 encoded protein not to react with ISAV antiserum, and it was concluded that it was either non-structural or a minor structural protein [12]. However, the fact that both segment 7 ORFs 1 and 2 products could be immunoprecipitated with antiserum against purified ISAV in the present study indicates that they are components of ISAV virions. We conclude from these experiments that (a) the four major polypeptides of 71 kDa, 53 kDa, 43 kDa, and 24 kDa, observed in gel electrophoresis of purified ISAV [16,17] correspond to products of segments 3, 5, 6, and 8-ORF2, respectively, and (b) only the products of segments 5, 6, and 7-ORF1 are glycosylated when expressed in presence of canine microsomal membranes, suggesting that the 7-ORF1 product could also be a membrane protein. The segments 5 and 6 products correspond to the two previously reported surface glycoproteins of ISAV, the F protein [9] and the HE protein [7], respectively.

### Further characterization of the protein encoded by segment 7 ORF 1

To determine if the segment 7 ORF 1 protein is one of the integral membrane proteins of ISAV, its amino acid sequence was examined for hydrophobic membrane attachment sequences or transmembrane motifs using the TMHMM prediction algorithm, version 2.0 [18], which showed it not to be a transmembrane protein. A similar observation was previously made by Ritchie *et al*. [10]. The sequence analysis of the segment 7 ORF 1 protein, however, revealed that it has four predicted N-myristoylation sites, at positions ^69^GSCVSF^74^, ^203^GLKGSW^208^, ^206^GSWGGW^211^, and ^271^GCTGSA^276^. Such a signal peptide(s) would allow the predicted protein to be associated with the membrane even in the absence of a transmembrane motif, making it a putative monotopic integral membrane protein [19].

**Figure 3:**
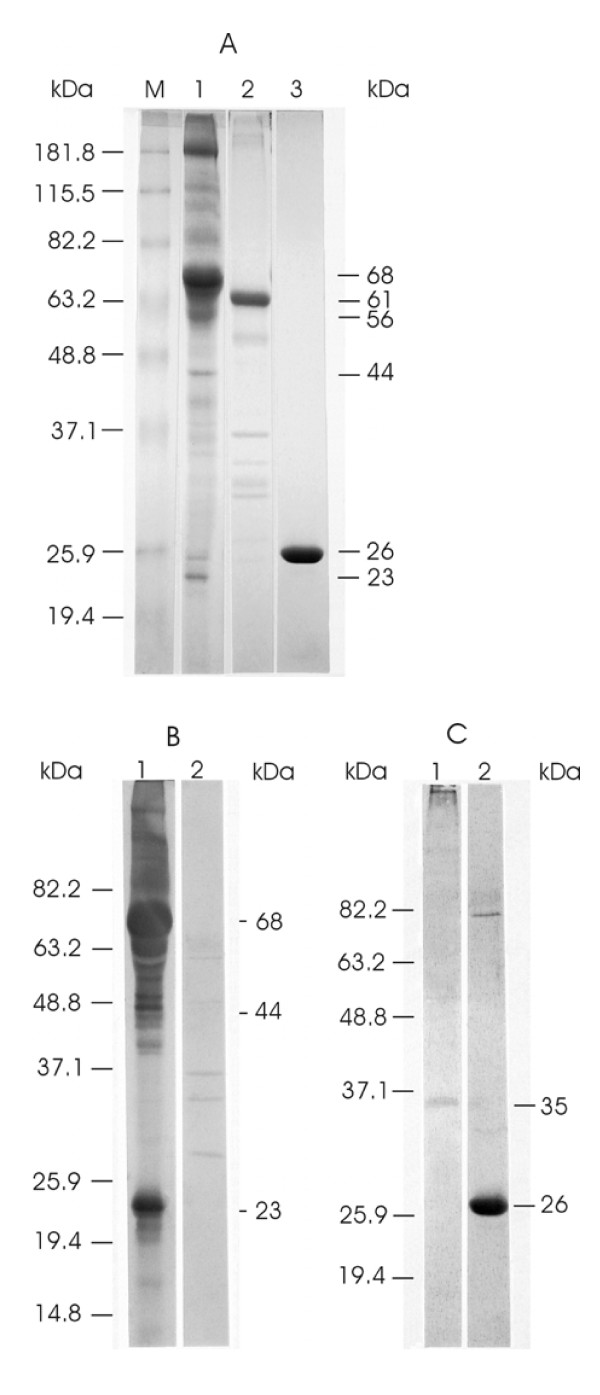
**Analysis of purified ISAV and GST proteins in Western blots**. The prestained protein standards (Invitrogen Life Sciences) are drawn in on the left while corresponding molecular masses of proteins are indicated on the right. **(A) **Coomassie blue-stained gel of the proteins. Lane M contains the prestained protein standards. Lanes 1 to 3 contain purified ISAV, GST-7ORF1 fusion protein inclusion bodies, and GST protein, respectively. **(B) **Western blots reacted with rabbit antiserum to whole ISAV. Lanes 1 and 2 contain purified ISAV and GST protein, respectively. **(C) **Western blots reacted with rabbit antiserum to GST-7ORF1 fusion protein. Lanes 1 and 2 contain purified ISAV and GST protein, respectively.

In order to more specifically characterize the segment 7 ORF 1 protein, a GST-fusion construct of segment 7 ORF1 (designated GST-7 ORF1) was expressed in *E. coli *for purposes of purifying it and using it to immunize a rabbit for preparation of antiserum, and for use in fish vaccination experiments. Expression in *E. coli *resulted in production of the GST-fusion protein in insoluble inclusion bodies despite the reduction of the incubation temperature to 15°C after IPTG (isopropyl-β-D-thiogalactoside) induction of bacterial cultures. Prior to use in the rabbit immunization and the fish vaccination experiments, the inclusion body fraction was extracted three more times with the lysis buffer, and was then resuspended in Arginine buffer. This buffer did not solubilize the inclusion bodies but prevented aggregation, allowing the inclusion bodies to remain in suspension for liquid handling. Both the rabbit polyclonal antiserum prepared against the GST-7 ORF1 fusion protein and the rabbit antiserum against whole purified ISAV were applied separately to Western blots of purified ISAV, GST-7ORF1, and GST protein alone. Figure 3A shows a Coomassie blue-stained gel of these samples. The rabbit antiserum to whole ISAV was specific for the purified virus (Fig. 3B, lane 1) and did not react with the GST protein (Fig. 3B, lane 2). The rabbit antiserum to GST-7ORF1 fusion protein reacted specifically with a single 35-kDa viral protein in Western blots of purified virus (Fig. 3C, lane 1), confirming that segment 7 ORF1 encodes a structural protein of ISAV. This antiserum also reacted with the GST protein (Fig. 3C, lane 2).

**Figure 4:**
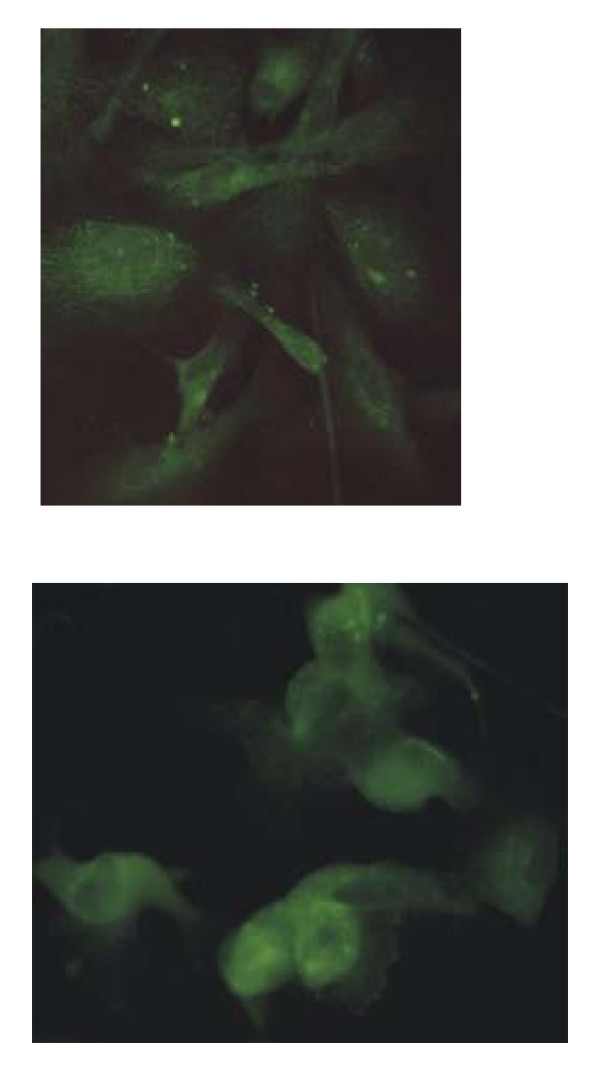
**Immunofluorescence of ASK-2 cell cultures infected with ISAV and stained with rabbit antiserum to GST-7ORF1 fusion protein**. Upper panel are cells 1 day post-inoculation with ISAV. Lower panel are cells 6 days post-inoculation with ISAV.

In order to demonstrate the sub-cellular localization of the segment 7 ORF1 protein, as well as to confirm the viral specificity of the rabbit antiserum prepared against GST-7ORF1 fusion protein, both uninfected and infected cell cultures (TO, ASK-2 and CHSE-214 cell lines) on slide flasks were fixed at 1, 3, and 6 days post-inoculation and used in immunofluorescence staining with the antiserum. The results on the infected cell monolayers are summarized in Table 2, and also presented in Figure 4, showing that the segment 7 ORF1 protein localizes in the cytoplasm. In contrast, no reaction was observed with the uninfected TO, ASK-2 and CHSE-214 cell samples (data not shown), indicating that the immunoreactive protein was of ISAV origin.

**Table 2:** Immunofluorescence of ASK-2 cell cultures infected with ISAV and stained with rabbit antiserum to GST-7ORF1 fusion protein.

Days post-inoculation with ISAV RPC/NB 980-049-1	ASK-2 cell line	TO cell line	CHSE-214 cell line
1	C-, IF±	C-, IF-	C-, IF-
3	C+++, IF+++	C+, IF+	C-, IF-
6	C++++++, IF++++++	C++++, IF+++++	C-, IF±

CPE (C)

Immunofluorescence (IF)

### Vaccination of Atlantic salmon with the GST-7 ORF1 fusion protein protects against ISAV-induced mortality

**Figure 5:**
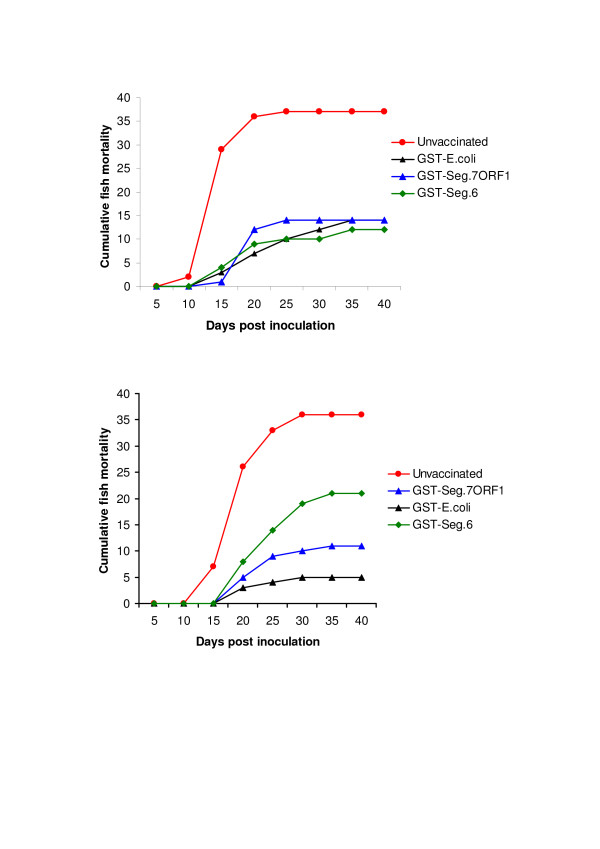
**Percent cumulative mortality of Atlantic salmon vaccinated with different vaccine preparations and then challenged with ISAV isolate NBISA01**. **(A) **Vaccinated fish challenged with low challenge dose (10^3.5 ^TCID_50_/0.2 ml/fish). **(B) **Vaccinated fish challenged with high challenge dose (10^6.0 ^TCID_50_/0.2 ml/fish).

In order to determine if the segment 7 ORF 1 protein is important in inducing protective immune responses in fish, a vaccine assay was performed. The vaccine preparations tested consisted of the following antigens emulsified in mineral oil (1) GST-7 ORF1 fusion protein in insoluble inclusion bodies, (2) GST-HE fusion protein in insoluble inclusion bodies, and (3) GST-*E. coli *lysate. In this particular study, the high challenge dose of 10^6.1 ^TCID_50_/fish was used to model protection against acute disease (i.e., ISA mortality starting between 10 and 13 days post-inoculation and lasting 9–15 days); and the low challenge dose of 10^3.5 ^TCID_50_/fish was used to model protection against chronic/protracted disease (i.e., ISA mortality lasting longer and/or starting later than in acute disease) [20]. Table 3 summarizes the relative percent survival (RPS), and Figure 5 shows the cummulative percent mortalities for the different vaccine preparations. The level of protection engendered by the GST-7 ORF1 fusion protein preparation was comparable to that of the GST-HE fusion protein preparation at the high challenge dose level. However, the GST-7 ORF1 fusion protein preparation resulted in a higher survival rate than what was attainable with the GST-HE fusion protein preparation, although it was only a moderate protection against the low ISAV challenge dose. Others who have tested recombinant ISAV HE vaccines have also reported only moderate protection resulting in 39.5–60.5% RPS after ISAV challenge [21]. Because only viral structural proteins are normally used as vaccine agents, we consider the immunizing properties of the GST-7 ORF1 fusion protein when administered as a vaccine in this study, as further evidence indicating that the segment 7-ORF1 product is a structural protein.

**Table 3:** Relative percent survival (RPS) of ISAV vaccines in Atlantic salmon using two different challenge doses

ISAV vaccine, and number of fish used	Total mortality (%)	**RPS^a^**
High challenge dose of 10^6.1 ^TCID_50_/fish		
GST-Seg 6, 46 fish	12 (26.1%)	67.5%
GST-Seg 7 ORF1, 46 fish	14 (30.4%)	62.2%
GST in BL21 *E. coli*, 45 fish	14 (31.1%)	61.3%
Unvaccinated fish, 46 fish	37 (80.4%)	0.0%
		
Low challenge dose of 10^3.5 ^TCID_50_/fish		
GST-Seg 6, 46 fish	21 (45.6%)	41.8%
GST-Seg 7 ORF1, 43 fish	11 (25.6%)	67.3%
GST in BL21 *E. coli*, 43 fish	8 (18.6%)	76.2%
Unvaccinated fish, 46 fish	36 (78.3%)	0.0%

^a^Calculation of RPS is an accepted method of determining the effectiveness of a vaccine [44]. RPS = [1-(% mortality of test group ÷ % mortality of control group)] × 100%.

### Characterization of ISAV segment 7 ORF1/2 protein

In a separate study in our laboratory, not described in this paper, the segment 7 ORF1/2 (18-kDa) product showed specific strong binding with caspase-8; also weaker binding reactions were noted with segments 1 ORF and 7 ORF1 products [22]. Analysis of the predicted amino acid sequence of the segment 7 ORF1/2 product in this study showed that it contains 3 leucine-rich segments (^44^LDLLRDQVDSL^54^, ^67^LDPGLYPWL^75^, ^113^LEFSLGL^119^). Similar sequences have been suggested to be essential for proper functioning of the influenza nuclear export protein (NEP) nuclear export signal [23], and leucine-rich nuclear export signals (LxxLxxLxLxL) have been described for other viral proteins such as HIV *Rev *[24]. We therefore speculate that the segment 7 ORF1/2 (18-kDa) protein is the putative ISAV NEP.

### Identification of a third functional ORF in ISAV segment 7

**Figure 6:**
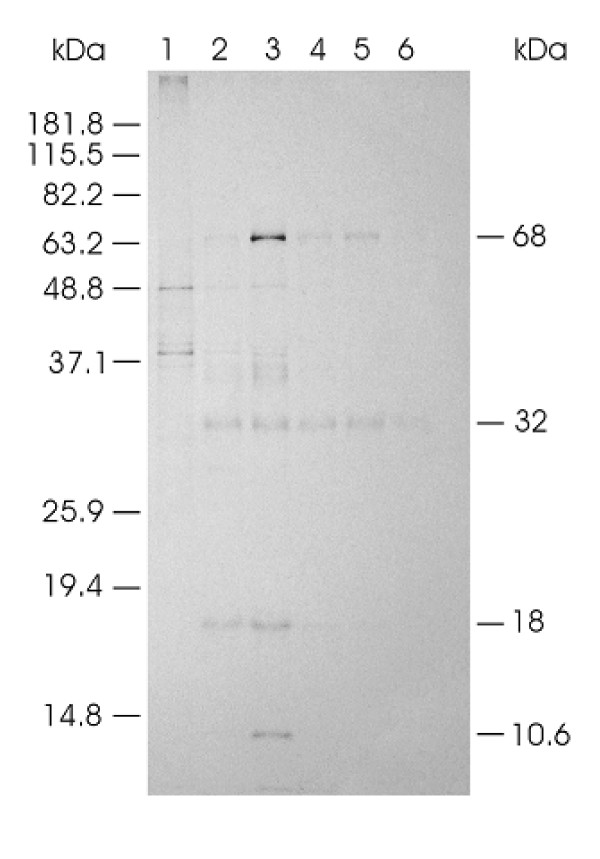
**Autoradiograph of SDS-PAGE of expressed proteins labeled with [^35^S]methionine in TO cells infected with ISAV, immunoprecipitated with rabbit antiserum to GST-7ORF1 fusion protein**. The prestained protein standards (Invitrogen Life Sciences) are drawn in on the left while corresponding molecular masses of immunoprecipitated ISAV proteins are indicated on the right. Lanes 1 to 6 contain radiolabeled proteins synthesized in uninfected TO cells, ISAV-infected TO cells at 1–5 days post-infection, respectively.

**Figure 7:**
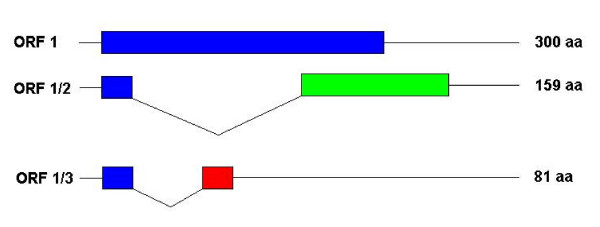
**New gene expression model for segment 7 of ISAV of North American genotype**. There are 3 ORFs consisting of the primary transcript (7-ORF1) 300 amino acids long, and 7-ORF1/2 and 7ORF1/3 each with an intron removed from 7-ORF1 and consisting of 159 and 81 amino acids, respectively.

The rabbit antiserum against the GST-7 ORF1 fusion protein was also used to directly monitor the synthesis of the 7-ORF1 protein in ISAV-infected cells in cell culture. For this, TO cell monolayers were infected with ISAV isolate RPC/NB 980-049-1 at a multiplicity of infection of 10 and individual flasks were removed daily from 1–5 days and were radiolabeled for 20 hr with 100 μCi of [35S]methionine. The cell monolayers were then harvested using RIPA buffer and were then subjected to immunoprecipitation with the rabbit antiserum against the GST-7 ORF1 fusion protein. Four individual protein bands of 68, 32, 18, and 10.6 kDa were detected in ISAV-infected cell lysates but not in uninfected cell controls (Fig. 6). The 68-kDa band corresponds to the ISAV NP protein [7]; cross-reactivity with the antiserum against GST-7 ORF1 fusion protein was surprising since there is no amino acid sequence homology between NP and 7-ORF1 proteins. It is possible that the two proteins have a similar conformation epitope. We have noted a similar cross-reactivity with a monoclonal antibody against the ISAV NP protein [25]. The 32-kDa protein was immunoprecipitated from lysates of the ISAV-infected TO cells throughout the sampling period (Fig. 6, lanes 2–6); the protein is comparable in size to that immunoprecipitated from ISAV-infected TO cell lysates using antiserum against purified ISAV (Fig. 2B, lanes 2–4). Thus the molecular mass of 35 kDa for the viral protein in the purified virus (Fig. 3C, lane 1) is larger than the segment 7 ORF1 in the infected cells and the *in vitro *transcription-translation product, and we consider this additional evidence for the post-translational modifications of the mature segment 7 ORF 1 protein. The 18-kDa protein was seen only at 1 and 2 days (Fig. 6, lanes 2–3) whereas the 10.6 kDa protein was present only at day 2 (Fig. 6, lane 3) of sampling. We speculate that these two proteins are also encoded by ISAV segment 7 and the cross-reactivity with the GST-7 ORF1 antiserum is because they share the N-terminal 22 amino acids of the 7-ORF1 protein. Previously, only the segment 7 ORF2 product was suggested to have this sequence [10], and would correspond to the 18-kDa protein in our study. We now show for the first time that a third protein of 10.6 kDa may have similar sequence. This spliced protein would consist of the first 22 amino acids of the 7-ORF1 product and a 257 nucleotide intron spliced out so that the translation continues in the +3 reading frame for a total of 81 amino acids (aa), and a predicted molecular weight of 9.5 kDa. This is a new putative ISAV protein never before reported, as a result of which we have also proposed a new gene expression model of ISAV segment 7 (Fig. 7), distinct from the coding strategy of segment 7 of influenza A virus. Analysis of 10 ISAV isolates for which we have full-length sequences of the 7-ORF1 gene, ISAV isolates RPC/NB 98-049-1, RPC/NB 02-1179-4, RPC/NB 98-0280-2, RPC/NB 02-0775-14, 7833-1, RPC/NB 01-0593-1, RPC/NB 01-0973-3, RPC/NB 04-085-1, 485/9/97, and 390/98 revealed that the segment 7-ORF1/3 polypeptide of 81 aa is present in all ISAV isolates of the North American genotype, whereas it terminates after 62 or 32 aa in ISAV isolates of the European genotype (Table 4).

**Table 4:** Open reading frames of RNA segment 7 of selected ISAV isolates

ISAV isolate	ISAV genotype^1^	GenBank Accession Number	Seg. 7-ORF1	Seg. 7-ORF1/2	Seg. 7-ORF1/3
RPC/NB 98-049-1	North American, HPR21	EF523761	1–903^2^	1–66/593–1006	1–66/324–503
RPC/NB 02-1179-4	North American, HPR21	EF523762	1–903	1–66/593–1006	1–66/324–503
RPC/NB 98-0280-2	North American, HPR20	AF401083	1–903	1–66/593–1006	1–66/324–503
RPC/NB 02-0775-14	North American, HPR21	DQ673253	1–903	1–66/593–1006	1–66/324–503
7833-1	North American, HPR21	AF401082	1–903	1–66/593–1006	1–66/324–503
RPC/NB 01-0593-1	North American, HPR21	EF523764	1–903	1–66/593–1006	1–66/324–503
RPC/NB 01-0973-3	North American, HPR21	EF523765	1–903	1–66/593–1006	1–66/324–503
RPC/NB 04-085-1	European, new HPR	EF432566	1–903	1–66/593–1006	1–66/324–446
485/9/97	European, HPR14	DQ673254	1–903	1–66/593–1006	1–66/324–362
390/98	European, HPR7	EF523763	1–903	1–66/593–1006	1–66/324–362

^1^Genotype of ISAV (unpublished results, F. S. B. Kibenge, M. J. T. Kibenge, Y. Wang, B. Qian, S. Hariharan, and S. McGeachy); HPR groups denote grouping based on amino acid sequences in the highly polymorphic region (HPR) of the ISAV haemagglutinin-esterase gene as reported by Nylund *et al*. [45] and Plarre *et al*. [46].

^2^Numbers refer to nucleotide positions of respective ORF.

## Conclusion

Several conclusions can be summarized: (i) Although a detailed analysis of all the ISAV proteins is not the primary subject of this communication, it is apparent that the ISAV genome, like the influenza A virus genome [26], encodes at least nine structural proteins but the gene order and putative functional identities of the ISAV proteins are significantly different from those of influenza viruses. (ii) We have provided a revised gene expression model of ISAV segment 7 together with evidence suggesting that segment 7 encodes 3 proteins. (iii) We have identified and characterized the protein encoded by segment 7 ORF1 as a 35-kDa minor structural protein of ISAV. (iv) Additionally, we show that vaccination of farmed Atlantic salmon with the segment 7 ORF1 protein resulted in a moderate protection against a severe ISAV challenge. (v) Based on the presence of nuclear export signals in the protein encoded by segment 7 ORF1/2, we have identified it as the putative ISAV nuclear export protein. (vi) The segment 7   ORF1/3 is a new protein of unknown function which is 81 amino   acids in ISAV of North American genotype but terminates after 62   or 32 aa in ISAV isolates of the European genotype. We are   presently studying these proteins to determine their functional roles   in ISAV.

## Methods

### Cells, viruses, and virus culture

TO cells [27] and ASK-2 cells [28] are continuous fish macrophage-like cell lines originally derived from Atlantic salmon head kidney leukocytes. TO cells were grown in HMEM [Eagle's minimum essential medium containing Hank's salts and sodium bicarbonate (0.2 g/L) BioWhittaker Inc., Walkersville, Maryland, USA] supplemented with 292 mM L-glutamine (Sigma), 1% non essential amino acids (Sigma), 100 μg ml-1 gentamicin (Sigma) and 10% fetal bovine serum (FBS). ASK-2 cells were grown in Leibovitz L-15 medium supplemented with 10% FBS, 2 mM L-glutamine, 100 IU/ml penicillin G, 100 μg ml-1 streptomycin, and 0.25 μg ml-1 amphotericin B. Both cell lines were incubated at room temperature (24°C) and the monolayer cultures were used 24 hrs after seeding. CHSE-214 cells [29] were grown at 16°C in HMEM [Eagle's minimum essential medium containing Hank's salts] (Invitrogen Life Technologies) as previously described [17]. The source and genotype of the ISAV isolates used in this study are summarized in Table 5. The viruses were propagated and titrated in TO cells as previously described [30] prior to use. The viruses were passaged once in ASK-2 cells or CHSE-214 cells before use in the respective cell lines.

**Table 5:** Origin and genotype of different ISAV isolates

Isolate and geographic origin	Laboratory source^1 ^and Year of isolation	ISAV genotype^2^
NBISA01, NB, Canada	Aqua Health, 1998	North American, HPR21
RPC/NB 98-049-1, NB, Canada	RPC, 1998	North American, HPR21
RPC/NB 98-0280-2, NB, Canada	RPC, 1998	North American, HPR20
7833-1, Chile	Aquatic Health, 1999	North American, HPR21
RPC/NB 01-0593-1, NB, Canada	RPC, 2001	North American, HPR21
RPC/NB 02-0973-3, NB, Canada	RPC, 20021	North American, HPR21
RPC/NB 02-0775-14, NB, Canada	RPC, 2002	North American, HPR21
RPC/NB 02-1179-4, NB, Canada	RPC, 2002	North American, HPR21
485/9/97, Norway	B. Dannevig, 1997	European, HPR14
810/9/99, Norway	B. Dannevig, 1999	European, HPR15
U5575-1, NS, Canada	AVC, 2000	European, HPR3
RPC/NB 04-085-1, NB, Canada	RPC, 2004	European, new HPR

^1^Sources of isolates were: Aqua Health Ltd, Charlottetown, PEI, Canada; RPC, Research and Productivity Council, Fredericton, NB, Canada; B. Dannevig, National Veterinary Institute, Oslo, Norway; A. McVicar, Fish Health Inspectorate, FRS, Marine Laboratories, Aberdeen, UK; Aquatic Health, Chile Ltd, Puerto Mont, Chile; AVC, Atlantic Veterinary College Regional Diagnostic Virology Laboratory.

^2^Genotype of ISAV (unpublished results, F. S. B. Kibenge, M. J. T. Kibenge, Y. Wang, B. Qian, S. Hariharan, and S. McGeachy); HPR groups denote grouping based on amino acid sequences in the highly polymorphic region (HPR) of the ISAV haemagglutinin-esterase gene as reported by Nylund *et al*. [45] and Plarre *et al*. [46].

### Construction of *in-vitro *transcription plasmids containing ISAV cDNA

The oligonucleotide primers (Table 1) spanning the ORFs in the eight RNA segments were designed from publicly available ISAV genome sequences in the GenBank database, and were purchased from a commercial source (Invitrogen Life Technologies). For segments 1, 2, and 3, overlapping RT-PCR products were initially generated and then used to obtain the complete ORFs. For the purpose of evaluating individual ORFs, we separated the otherwise partial overlapping ORFs of segment 7 based on the gene expression model suggested by Ritchie *et al*. [10], of partial splicing of ORF1 mRNA and a frameshift such that ORFs 1 and 2 products share the first 22 amino acid residues. The RT-PCR amplification of viral cDNA and cloning and nucleotide sequencing procedures used in this study were previously described [30]. Thus the PCR products were cloned into the pCR^®^II vector having dual promoters T7 and SP6 in opposite orientation (Invitrogen Life Technologies) for *in vitro *coupled transcription and translation (TNT-Promega) reactions. Plasmids were sequenced to check for correct orientation and to verify each viral cDNA sequence.

### Cell-free transcription-translation

The pCR^®^II-TOPO^® ^recombinant plasmids were used as circular DNA templates. Approximately 1 μg each of plasmid DNA was transcribed directly in a translation mix containing 20 μCi of [35S]methionine (Amersham Pharmacia Biotech) in 50 μl-reaction volumes using the *in vitro *transcription-translation TNT™ T7/SP6 Coupled Reticulocyte Lysate System (TNT-RLS, Promega) as per the manufacturer's instructions. A Luciferase DNA (supplied by Promega) was used as positive control. Following incubation of the reactions for 90 min at 30°C, 5 μl of each reaction were mixed with 20 μl of 2× Laemmli sample treatment buffer (0.125 M Tris-HCl pH 6.8, 4% SDS, 20% glycerol, 0.002% bromophenol blue, 4% 2-mercaptoethanol) [31] and heated at 60°C for 20 min to denature the proteins. Ten microliters of the denatured sample were resolved on discontinuous SDS-12-5% PAGE [32] and radiolabeled proteins in dried gels were visualized by autoradiography for 48 h at room temperature. The molecular weights of the proteins were calculated from the gels using standard procedures [33]. To examine for glycosylation, a general protocol recommended by the manufacturer for translation in the TNT-RLS with canine microsomal membranes (Roche Molecular Biochemicals) was followed and samples were analyzed by 12.5% SDS-PAGE and autoradiography.

### Construction of pGEX expression plasmid and preparation of GST fusion protein

The pCR^®^II-TOPO^® ^recombinant plasmids, pCR TOPO-Seg7ORF1 and pCR TOPO-Seg6ORF were used as the source of the ISAV segment 7 ORF1 and segment 6 ORF, respectively. PCR was performed using the DeepVent DNA polymerase (New England Biolabs) as specified in the manufacturer's protocol. The PCR products were subcloned into the pGEX-5X-2 expression vector (Amersham Biosciences) to produce constructs pGEX5X2-7ORF1 and pGEX5X2-6ORF. These constructs were designed to generate the full-length ISAV segment 7 ORF1 polypeptide and the ISAV HE polypeptide, respectively, each fused with GST at its N-terminus, and this was verified by DNA sequencing.

The plasmid constructs were used to transform competent *E. coli *strain BL21(DE3)(pLysS) (Novagen) in order to express the GST fusion proteins. Transformants were cultivated on LB (Luria-Bertani) broth agar medium containing 100 μg ml-1 ampicillin. An overnight LB broth culture of the transformant was pelleted by centrifugation, washed with LB medium (10 g/L bactotryptone, 5 g/L yeast extract, and 5 g/L NaCl, adjusted to pH 7.4) containing 200 μg ml-1 ampicillin and diluted 100× in 2-4L of LB medium supplemented with 2 g/L dextrose and 150 μg ml-1 ampicillin and grown at 37°C with agitation (165 rpm). When the optical density reached a value of 0.7 at 600 nm, protein expression was induced at 15°C by addition of IPTG (Amersham Biosciences) to a final concentration of 0.1 mM. Cells were harvested by centrifugation at 6,000 *g *for 12 min at 4°C and resuspended in 40 ml PBS (150 mM NaCl, 16 mM Na_2_HPO_4_, and 4 mM NaH_2_PO_4_, pH 7.3). This step was repeated twice to wash the cells. The supernatant was discarded and the wet cell paste was frozen at -20°C overnight. The bacterial cells were thawed, re-suspended in lysis buffer containing 50 mM Tris pH 8.0, 500 mM NaCl, 1 × BugBuster protein extraction reagent (Novagen), 1 mM DTT, 10% glycerol, 300 μg ml-1 lysozyme, 1 mM PMSF, Complete protease inhibitor (Roche Applied Science), and incubated with stirring for 2 h at 4°C. Then, Benzonase nuclease (Novagen) was added as specified by the supplier, incubated with slow stirring at 4°C for 1 h. The cells were centrifuged at 10,000 *g *for 1 h. The supernatants were collected and filtered using 0.45 μm filters (Gelman Sciences) for affinity chromatography purification. The GST-7ORF1 fusion protein and the GST-HE fusion protein contained in the supernatants were separately purified by affinity chromatography using Glutathione Sepharose (Amersham Biosciences) batch method at 4°C according to the manufacturer's protocol. The final elutes of each fusion protein were pooled, concentrated and desalted using Centricon-Plus 20 (Amicon). The concentration of each fusion protein was estimated roughly by spectrophotometry at 280 nm where 1A = 0.5 mg/ml GST fusion protein, and its purity and integrity were analyzed by resolving it on discontinuous SDS-12.5% PAGE [32] and visualized with Coomassie Blue R-250 (Bio-Rad). The BL 21 strain of *E. coli *without ISAV gene was lysed with 0.5% NP-40 (Sigma) and then clarified at 700 *g *to obtain the bacterial lysate without ISAV proteins.

### Rabbit antiserum production

Preparation of rabbit polyclonal antiserum to purified ISAV isolate RPC/NB 980 049-1 has been described [15]. Rabbit polyclonal antiserum to GST-7 ORF1 fusion protein was prepared using a similar procedure. Briefly, GST-7 ORF1 fusion protein inclusion bodies were resuspended in Arginine buffer (10 mM Tris-HCl, 150 mM NaCl, 800 mM L-Arginine, pH 8.5). Two hundred and fifty microliters of the suspension were brought to 1.25 ml with PBS and were then aliquoted into 250 μl-volumes. One 250 g rabbit (Charles River Canada) was inoculated subcutaneously five times at 3-week intervals with 250 μl of the GST-7 ORF1 fusion protein, once mixed with an equal volume of Freund's complete adjuvant and four times in Freund's incomplete adjuvant. Pre-inoculation serum and sera collected every 3 weeks until after the last injection were tested for the presence of antibodies against purified ISAV by Western blot analysis.

### Fish vaccination assay

Specific pathogen-free Atlantic salmon parr (St. John River stock) were obtained either from the Cardigan Fish Hatchery, Cardigan, P.E.I. or Atlantic Sea Smolt Ltd., Souris, P.E.I., Canada. The average weight of the fish at the start of the trial was 9.1 gm in the freshwater phase. An opportune sample of five fish was screened for ISAV by virus isolation attempts from tissue samples (kidney, heart, spleen, pyloric caeca) on the TO cell line and monitoring for CPE and RT-PCR [30] to establish the ISAV-negative status of the stock before the vaccination. The fish were maintained in the Aquatic Animal Facility of the Atlantic Veterinary College (AVC) in 1 m diameter fibreglass-reinforced plastic tanks using a freshwater flow through system at a temperature of approximately 11°C. The fish were acclimatized in freshwater for 2 weeks at a water flow rate of 3.5 L/min. The experimental procedures used in this study were performed in accordance with the guidelines of the Canadian Council of Animal Care [34].

The fish were divided into groups: the unvaccinated group consisted of 187 fish in one tank while each of the vaccinated groups consisted of 43–46 fish per tank in two tanks. For the vaccination, the fish were removed from the stock holding tank and anaesthetized by immersion in an aerated solution of tricaine methane sulphonate (TMS-222) (100 mg/L). The vaccination consisted of an intraperitoneal injection with a vaccine dose of 50 μg/0.2 ml/fish and the fish was then returned to the respective study tank. The fish were held for 56 days and then each vaccinated group was challenged with ISAV isolate NBISA01 using two different doses for each two tanks; a high dose (10^6.1 ^TCID_50_) and a low dose (10^3.5 ^TCID_50_). For this challenge trial, the fish in the unvaccinated tank were divided into three groups: two groups consisted of 46 fish in each tank to serve as the unvaccinated challenged controls for the high and low challenge doses while the remaining 95 fish were moved to an uninfected control tank which was located in a separate "clean" room away from the tanks with the vaccinated fish. For the experimental infection, the challenge fish were removed from the respective tank and anaesthetized as before. Each fish was then challenged by the intraperitoneal route with 0.2 ml of virus suspension of the high dose or low dose of ISAV and was then returned to the respective study tank. Caution was taken to avoid contact between the tanks for the two different challenge doses. Fish were fed once everyday and the fish tanks were flushed once a day. All tanks were checked twice daily for mortality, the fish were observed for abnormal behaviour, and external lesions until the study was terminated at 42 days post challenge. All dead fish were necropsied and, where necessary, tissues (kidney, heart, spleen, pyloric caeca) were collected for virus detection by reverse transcriptase PCR (RT-PCR) amplification and histopathology.

### Pulse labeling infected cells with [^35^S]methionine

The TO, ASK-2, and CHSE-214 cells in 4-well tissue culture plates or 25 cm^2 ^tissue culture flasks were infected with ISAV at a multiplicity of infection of 10, and labeled with [^35^S]methionine as described previously [15,35], with slight modifications. Briefly, at 1, 2, 3, 4, 5, and 6 days post-infection, monolayers were washed twice with cold phosphate buffered saline (PBS), pH 7.2, and were incubated in 1 ml/well methionine-free EMEM supplemented with 5% dialyzed FBS to deplete residual methionine. After 2.5 h, cells were radiolabeled for 20 h with 100–200 μCi of [35S]methionine (specific activity approximately 1000 Ci/mmol at 10 μCi/μl; Amersham Pharmacia Biotech) in 1–2 ml methionine-free EMEM containing 5% dialyzed FBS. To obtain labeled cells, the medium was aspirated and cells were washed twice with PBS and then lysed with 500 μl RIPA buffer (150 mM NaCl, 1% sodium deoxycholate, 1% aprotinin, 1% Triton X-100, 1% sodium dodecyl sulfate, 10 mM Tris-HCl, pH 7.2). The lysate was centrifuged at 12,000 *g *for 2 min and the supernatant was transferred to a fresh microfuge tube and stored at -20°C until used.

### Immunoprecipitation of polypeptides

ISAV proteins synthesized in ISAV-infected cells or by *in vitro *transcription-translation system were analyzed by immunoprecipitation with rabbit antiserum to purified ISAV isolate RPC/NB-980 049-1 prepared as described previously [15]. The immunoprecipitation was carried out as described by Sambrook *et al*. [36], with slight modifications. Briefly, the 10 μl of cell lysates or in vitro translation sample brought to 500 μl total volume in NET-gel buffer (150 mM NaCl, 0.1% Nonidet P-40, 1 mM EDTA, pH 8.0, 0.25% gelatin, 0.02% sodium azide, 50 mM Tris-HCl, pH 7.5) were initially precleared with pre-immune rabbit serum and Protein-A Sepharose beads (Sigma) to reduce non-specific background. For the specific immunoprecipitations, each precleared supernatant was reacted with 20 μl of rabbit hyper-immune antiserum at 0°C for 1 h. Each mixture was then reacted with Protein-A Sepharose beads at 4°C for 1 h. The Sepharose beads were washed with 10 mM Tris-HCl, pH 7.5, 0.1% NP-40, mixed with 30 μl of 2× SDS sample loading buffer, and heated at 100°C for 5 min prior to fractionation on discontinuous SDS-12-5% PAGE [32]. Radiolabeled proteins in dried gels were visualized by autoradiography for 48 h at room temperature.

### Gel electrophoresis of viral proteins and Western blot analysis

Samples were mixed with an equal volume of 2× SDS sample loading buffer, and heated at 100°C for 5 min prior to fractionation on discontinuous SDS-12-5% PAGE (Laemmli 1971). Proteins were visualized with Coomassie blue R-250 (Bio-Rad). Duplicate gels were used in Western blotting as described previously [17]. Briefly, resolved proteins in unstained gels were transferred from the gel to the nitrocellulose membranes using a Trans-Blot apparatus (Bio-Rad) for 4 hr at 60 V (65 mA/cm^2^). The membranes were blocked with PBS containing 3% skim milk and then reacted with a 1:50 dilution of rabbit antiserum to GST-7 ORF1 fusion protein or to purified ISAV isolate RPC/NB-980 049-1. The blots were reacted with a 1:30,000 dilution of goat anti-rabbit alkaline phosphatase conjugate (Sigma) and colour was developed with BCIP/NBT substrate (Bio-Rad).

### DNA sequencing and analysis of sequence data

Plasmid DNA for sequencing was prepared as described before [37]. Denatured plasmid DNA was sequenced using the ABI BIG DYE terminator version 3.1 cycle sequencing kit (Applied Biosystems, Inc.) and the MJ Research thermal cycler. Sequencing reactions were resolved on two DNA sequencers. The first, a model 377 ABI Prism Automated DNA Sequencer (Applied Biosystems, Inc.) using 36–64 lanes on a 48 cm plate. ABI's 'mobility file' that comes with the dye terminator kit was used to correctly call the bases. The electropherograms were inspected and edited using "Sequencing Analysis 3.3" software provided with the 377 Prism by ABI. The second, a model 3100 ABI Genetic Analyzer (Applied Biosystems, Inc.) with a 50 cm – 16 capillary array using POP-6 polymer. ABI's 'mobility file' that comes with the dye terminator kit was used to correctly call the bases. The electropherograms were inspected and edited using "Sequencing Analysis 5.1.1" software provided with the 3100 ABI Genetic Analyzer. Sequence analysis used the BLAST programs [38], the Sequence Manipulation suite [39], and the FASTA program package for microcomputers [40]. The following programs available through ExPASy Molecular Biology Server [41] were also utilized: ScanProsite, MotifScan, PROSCAN, SignalP. V1.1, and TMHMM server v. 2.0 [18,42]. The ProtScale program was accessed via the Sanger web site [43].

## Competing interests

The author(s) declare that they have no competing interests.

## Authors' contributions

FSBK conceived the study, coordinated the research efforts, performed the cell-free transcription-translation, pulse labeling and immunoprecipitation experiments and sequence analysis, prepared the rabbit antiserum to GST-7ORF1 fusion protein, prepared the fish vaccines and challenge virus and drafted the manuscript. HX prepared the pGEX expression plasmids and GST fusion proteins, performed the immunofluorescence assays, the sequence analysis, and helped to draft the manuscript. MJTK made plasmid DNA isolations, conducted the fish vaccination assay and assisted with writing the manuscript. BQ prepared the *in-vitro *transcription plasmids containing ISAV cDNA and performed the Western blot analysis. TJ assisted with the plasmid DNA isolations, and conducted experiments on segment 7 ORF1/2 protein. All co-authors read and approved the final manuscript.
